# Supplementary material for: MOC Composites for Construction: Improvement in Water Resistance by Addition of Nanodopants and Polyphenol
Source: Polymers (Basel). 2023 Nov 1;15(21):4300. doi: 10.3390/polym15214300 (PMC10650835; doi:10.3390/polym15214300)
Supplement: Supplementary file 1 [file polymers-15-04300-s001.zip › polymers-2687919-supplementary.pdf]

polymers MDPI

SI – Supplementary Information

Experimental details

The phase composition of the samples was studied using X-ray powder diffraction (XRD). The data were collected at room temperature on Bruker D8 Phaser (Bruker, Germany) powder diffractometer with parafocusing Bragg–Brentano geometry using CuKα radiation (λ = 0.15418 nm, U = 30 kV, I = 10 mA). Data were scanned over the angular range 5–80° (2θ) with a step size of 0.019° (2θ) and evaluated in the X'Pert HighScore Plus software. For each measurement approximately ~1 g of crushed sample was used.

The microstructure and morphology of the prepared samples was investigated using scanning electron microscopy (SEM) with a FEG electron source (Tescan Lyra dual-beam microscope). The study of the elemental composition and the elemental mapping were performed using an energy dispersive spectroscopy (EDS) analyzer (X-MaxN) with a 20 mm^2 SDD detector (Oxford instruments) and AZtecEnergy software. The samples were crushed and small pieces of them were placed on an adhesive carbon conductive tape. The samples were then covered with a 10 nm layer of gold using a sputtering technique. The SEM and SEM-EDS measurements were carried out using a 10 kV electron beam.

Samples aged for 28 days were used to measure macrostructural, microstructural and mechanical parameters. Among the basic material characteristics, bulk density ρ_b ($kg{\cdot}m^{-3}$), matrix density ρ_{mat} ($kg{\cdot}m^{-3}$) and total open porosity φ (%) were tested. The expanded combined uncertainty of the bulk density determination was 1.4%. The matrix density was measured using a Pycnomatic ATC helium pycnometer (Thermo Scientific). The expanded combined uncertainty of this test was 1.2%. The total open porosity was obtained based on the knowledge of the bulk and specific density values. The expanded combined uncertainty of the total open porosity determination was 2.0%.

The flexural strength f_f (MPa) testing was conducted in a three-point bending test arrangement on a Heckert PF 100 mechanical press. The specimen fragments from the flexural strength test were used to evaluate the compressive strength f_c (MPa). The loading area in the uniaxial compressive strength test was 40 mm × 40 mm. Both strength tests were performed according to with EN 1015-11 [72]. The dynamic modulus of elasticity E_d (GPa) was determined in the ultrasonic velocity test using a Vikasonic apparatus (Schleinbinger Geräte). The expanded combined uncertainty of both strength tests was 1.4% and that of the ultrasonic velocity test 2.3%, respectively.

The 24-h water absorption W_{a24} ($kg{\cdot}m^{-3}$) by immersion at atmospheric pressure was obtained. The samples were 40 mm cubes insulated by epoxy resin on all lateral sides (to ensure 1D water transport) and were immersed for 24 hours in a tank filled with tap water. Based on the measurement sample mass and its volume, W_{a24} was calculated. The expanded combined uncertainty of the water absorption assessment was 1.2%.

As durability parameter, softening coefficient s_c (-) was assessed as a ratio of the 28-day compressive strength of control samples stored at laboratory conditions and that of the samples immersed for 24 h in water.

References

72. EN 1015-11, Methods of test for mortar for masonry - Part 11: Determination of flexural and compressive strength of hardened mortar, CEN, Brussels, Belgium, 1999.
